# The feasibility of a novel 3D-Printed patient specific cutting guide for extended trochanteric osteotomies

**DOI:** 10.1186/s41205-024-00204-3

**Published:** 2024-03-01

**Authors:** Reza Bergemann, Gregory R. Roytman, Lidia Ani, Alim F. Ramji, Michael P. Leslie, Steven M. Tommasini, Daniel H. Wiznia

**Affiliations:** 1 Orthopaedics and Rehabilitation, Yale School of Medicine, Yale University, 333 Cedar St. FMB 5, New Haven, CT 06511 USA; 2 Biomedical Engineering, Yale School of Engineering and Applied Sciences, Yale University, New Haven, USA; 3 Mechanical Engineering and Material Sciences, Yale School of Engineering and Applied Science, Yale University, New Haven, CT USA

**Keywords:** 3D Printing, Cutting guide, Total hip arthroplasty, Extended trochanteric osteotomy, Patient specific instrumentation

## Abstract

**Background:**

The extended trochanteric osteotomy (ETO) is a surgical technique utilized to expose the intramedullary canal of the proximal femur, protect the soft tissues and promote reliable healing. However, imprecise execution of the osteotomy can lead to fracture, soft tissue injury, non-union, and unnecessary morbidity. We developed a technique to create patient specific, 3D-printed cutting guides to aid in accurate positioning of the ETO and improve osteotomy quality and outcomes.

**Methods:**

Patient specific cutting guides were created based on CT scans using Synopysis Simpleware ScanIP and Solidworks. Custom 3D printed cutting guides were tested on synthetic femurs with foam cortical shells and on cadaveric femurs. To confirm accuracy of the osteotomies, dimensions of the performed osteotomies were compared to the virtually planned osteotomies.

**Results:**

Use of the patient specific ETO cutting guides resulted in successful osteotomies, exposing the femoral canal and the femoral stem both in synthetic sawbone and cadaveric testing. In cadaveric testing, the guides allowed for osteotomies without fracture and cuts made using the guide were accurate within 6 percent error from the virtually planned osteotomy.

**Conclusion:**

The 3D-printed patient specific cutting guides used to aid in ETOs proved to be accurate. Through the iterative development of cutting guides, we found that a simple design was key to a reliable and accurate guide. While future clinical trials in human subjects are needed, we believe our custom 3D printed cutting guide design to be effective at aiding in performing ETOs for revision total hip arthroplasty surgeries.

## Introduction

**Fig. 1:**
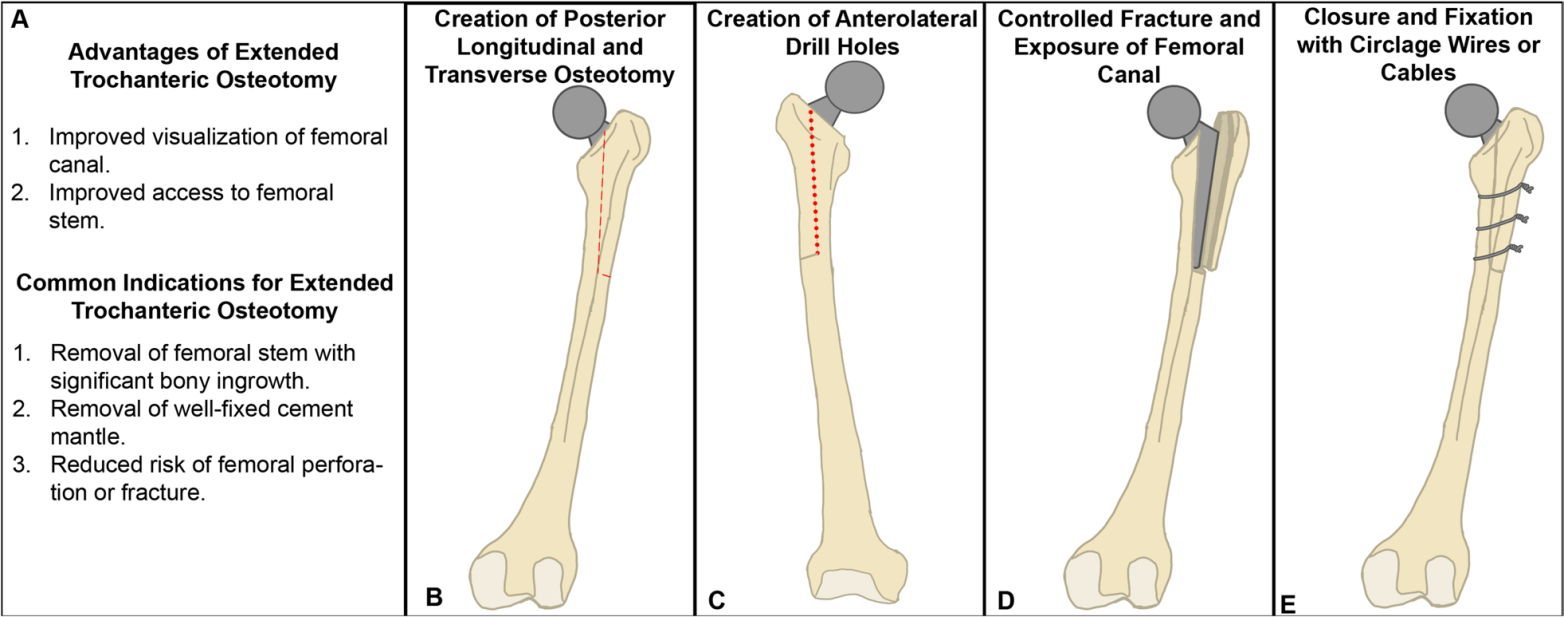
Overview of the Extended Trochanteric Osteotomy. **A** Overview of the advantages and indications for the extended trochanteric osteotomy. **B–E** Basic overview of the steps involved in a traditional extended trochanteric osteotomy

Extended trochanteric osteotomy^1^ (ETO) (Fig. 1) is a surgical technique that can be performed in revision total hip arthroplasty (rTHA) to safely improve visualization of the intramedullary canal and access to the femoral stem. The procedure was popularized in the 1990s to effectively address challenges that may arise during rTHA, such as well-fixed cement or extensive bony in-growth of the femoral component [1, 2]. An ETO can provide improved exposure to facilitate removal of a femoral stem that may be challenging due to factors as mentioned above, and can reduce the risk of femoral perforation or fracture [3–5].

Union rates as high as 98.7% have been reported for ETOs, suggesting reliable healing potential [4]. Despite the described advantages of ETOs, the procedure is technically challenging, with an average operative time of 165 min and estimated blood loss range between 600–2500 mL [6]. Furthermore, ETOs may result in morbidity such as intraoperative fractures, soft tissue injury and nonunion [7, 8]. These fractures often involve the greater trochanter therefore creating a segmental lateral cortex that is both technically difficult to stabilize and subsequently heal. In addition, there is a lack of precision involved in determining the most optimal location of the osteotomy, including the distal extent of the osteotomy. Surgeons typically plan the length of their osteotomies from measurements taken from preoperative x-rays or utilize fluoroscopy to localize the distal extent of the stem. Therefore, a more precise and customized osteotomy has the potential to further decrease surgical time and minimize complications.

Over the past few years, 3D printing, an additive manufacturing method capable of efficiently producing complex geometries, has found many new uses in personalized medicine. It has been used to facilitate surgical planning and patient education through the creation of anatomical models [9–11] and in orthopaedic surgery in particular [12]. 3D printing has been used to produce patient specific instrumentation (PSI) such as cutting guides [13, 14], drilling guides [15–17], and custom implants for atypical anatomical areas that are both safe and effective [18, 19]. These instruments have been found to improve accuracy, shorten operative time, decrease intraoperative imaging requirements [13] and reduce intraoperative blood loss [20]. Given that ETOs are complex patient specific procedures, we hypothesized that patient specific cutting guides for ETOs using 3D printing would be accurate with respect to virtual planning of intended cuts.

## Methods

This research involved decedents without personal identifiers so it is not considered human subjects research and does not require IRB review and approval. Fresh-frozen cadaveric samples were handled appropriately in accordance with supplier instructions and site regulation.

### Surgical guide design and fabrication

**Fig. 2:**
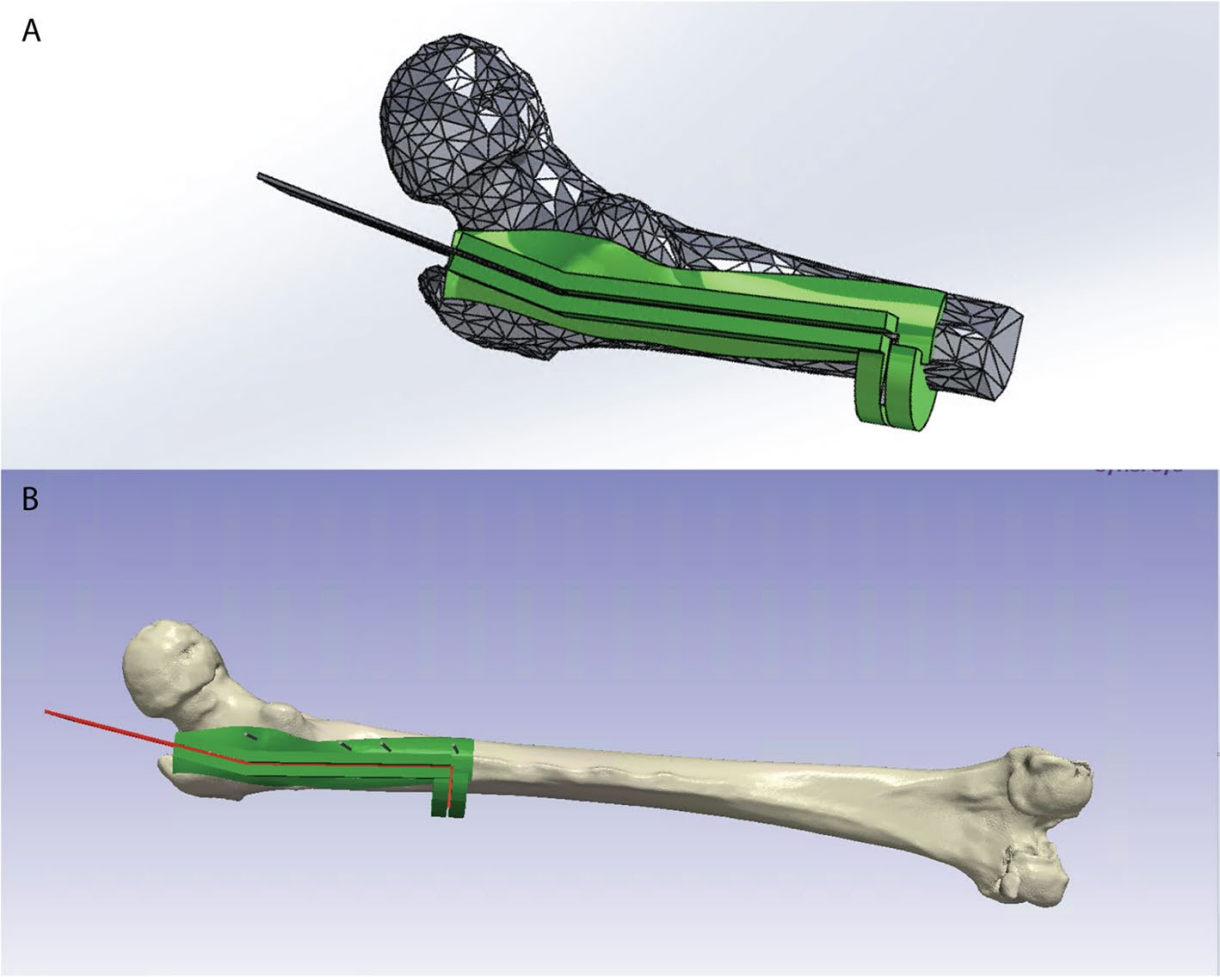
Virtual modeling of surgical guide and cadaveric femur. a. Low-resolution model of femur (grey) and cutting guide (green) as designed in Solidworks. b. Segmented femur (white) with finalized cutting guide (green), cutting planes (red) and K-wires (grey) as modeled in ScanIP

CT scans were obtained for both the synthetic sawbone femur models (Sawbones, Femur, 15 mm Canal, Solid Foam, Left, Medium) and cadaveric femurs prior to surgical planning and guide development. CT scans were obtained using LightSpeed VCT GE Medical Systems Computed Tomography Scanner with a slice thickness of 0.625 mm and kVp of 80 and 140 for the sawbones and cadaveric femurs respectively. Using Synopysis Simpleware ScanIP (Version T-2022.03-SP2, 2022, Mountain View, CA, USA) image processing software to segment CT scans, 3D models of the femur were generated. For cadavers with total hips implanted, 3D models of the implants were generated as well. The osteotomies were modeled as 1.2 mm planes to account for the width of the sawblade and planned and positioned in consultation with adult reconstruction orthopaedic surgeons with more than five years of post-fellowship experience. Virtual models of the femur and osteotomies were then imported as reference geometry into Dassualt Systèmes SolidWorks (Version 2021–2022, Vélizy, France) and used to design the surgical guide (Fig. 2). Once a 3D model of the cutting guide was generated, the model was brought into ScanIP once more for the final planning. In ScanIP, 1.6 mm Kirshner wires (K-wires), modeled as simple cylinders, were placed to aid in fixing the cutting guide to the femur. The topology of the femur and the K-wire was subtracted from the surgical guide model, so that the guide would fit tightly onto the surface of the femur. The overall geometry of the surgical guide was developed to respect the soft tissue as much as possible and was designed to conform primarily to the exposed bone on the posterior surface, requiring minimal stripping of vastus lateralis soft tissue from the bone and protecting the gluteus medius insertion.

Prior to 3D printing, the finalized design was reviewed by an orthopaedic surgeon in ScanIP. ScanIP enabled the surgeon to view the surgical guide, osteotomy locations and the femur and femoral stem in three dimensions. The final model of the cutting guide was printed on a FormLabs Form 3BL stereolithography SLA 3D printer. This 3D printer utilizes inverted vat polymerization, which enables quick and cost effective printing, with the high level of detail required for personalizing the cutting guides to patient anatomy. Prints were oriented so that supports were attached to the external surfaces of the guides. This was important to ensure that the supports did not interfere with the cutting guides ability to conform to the cortical bone of the femur. All surgical guides were post-processed following material guidelines provided by the manufacturer; the printed guides were washed in isopropyl alcohol for 10 min, before UV cured for a minimum of 30 min.

There were several design iterations that led to continuous improvement of the cutting guide. For the first iteration of the design, the 3D model of the cutting guide was evaluated in ScanIP and was determined to likely interfere with the soft tissue not visible on the CT scan. A soft tissue sparing second iteration was created, though upon 3D printing of the guide, it was found to be too bulky and too flexible, and thus would not function accurately.

A third iteration design was then developed with increased rigidity while respecting soft tissue, and was printed using FormLabs Grey resin. The guide was tested on a synthetic sawbone femur models (Sawbones, Femur, 15 mm Canal, Solid Foam, Left, Medium). In this sawbones testing, the antero-lateral drill-guide portion was found to be unnecessary, and was eliminated in the final iteration.

The fourth iteration design was tested on three cadaveric femurs. All three cadaveric femurs had soft tissue. Only Cadaver 1 had a femoral stem in place. Cadaver 2 had both Left and Right femurs without femoral stems, and the surgery was planned as if there was a femoral stem in place. The guide for Cadaver 1 was printed using FormLabs Grey and the guides for Cadaver 2 Left and Right were printed using FormLabs Tough 2000 and FormLabs Biomed Amber, respectively. Since Cadaver 2 Right was printed using Biomed Amber resin, a resin that is approved as biocompatible and for surgical use, the guide was sterilized using an autoclave for 30 min to simulate the stresses the guide might undergo during the sterilization process.

### Testing of surgical guides

All testing was performed by an orthopaedic surgeon five years post fellowship training. The first two iterations were qualitatively evaluated in ScanIP to evaluate any potential issues. The third iteration surgical guide was tested on a synthetic femur sawbone model with foam cortical shell. The ETO approach involved a posterior longitudinal osteotomy, a transverse osteotomy, as well as anterolateral drill holes. For these procedures, the cutting guide conformed stably to the femoral geometry, allowing for accurate positioning. K-wires were then inserted using a wire driver, fixing the guide to the femur. Next, an oscillating sagittal saw was used to make the longitudinal and transverse osteotomies as directed by the cutting guide. Then, a pencil tipped burr was used to make the anterolateral drill holes. Finally, osteotomes were used to create a controlled fracture connecting the posterior osteotomy to the anterolateral holes and lever the osteotomy open, exposing the femoral canal and implant.

While the third iteration surgical guide resulted in a successful ETO, the anterolateral drill guide portion was found to be unnecessary as the posterior guide provided a trajectory that sufficiently accommodated the anterior cortical osteotomy. A fourth-iteration guide was developed that utilized the trajectory of the posterior osteotomy to also serve as the trajectory of the anterior osteotomy by having the saw blade cut through the posterior cortex, skim over the lateral aspect of the femoral implant, and then cut through the anterior cortex. The fourth iteration was designed to conform to the geometry of the model femur and to be fixed with K-wires. This fourth iteration was tested on three cadaveric hips with soft tissue, with an orthopaedic surgeon performing the procedure. For each femur, a custom guide was produced. For the initial cadaveric test, three versions of the surgical guide were produced with varying amounts of offset from the femur to account for the soft tissue.

**Fig. 3:**
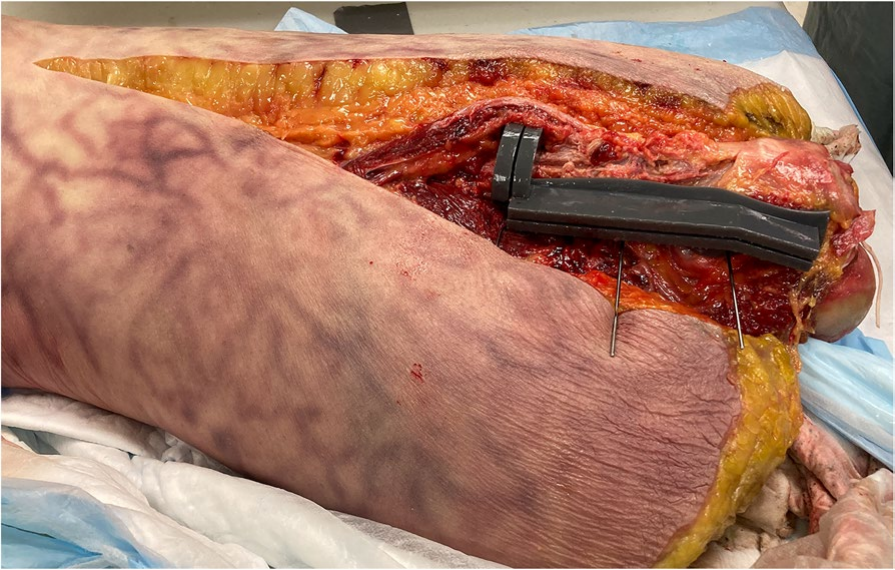
Attachment of surgical guide to the exposed Cadaver 2 Left femur with K-wires

Utilizing a posterior approach, each cadaver was dissected, starting with a curvilinear incision centered over the lateral aspect of the greater trochanter. The fascia lata was incised, and the soft tissue was then retracted to visualize the femur. A minimal amount of vastus lateralis needed to be elevated to allow the 3D printed guide to fit. The cutting guide was then fixed with K-wires and an oscillating saw was used to create the posterior osteotomy. The saw blade skimmed over the lateral aspect of the femoral stem, and an anterior cortical osteotomy was conducted. A transverse distal cut was then made, and the osteotomy was levered open with flat osteotomes (Fig. 3). Finally, the osteotomy was measured for comparison to the planned procedure, and error was calculated based on measurements made in ScanIP.

## Results

### Design iteration and sawbone femur testing

Our initial design focused on guiding the posterior longitudinal and transverse osteotomies, as well as anterolateral drill holes. To accurately align the surgical guide to the femur, the lesser trochanter and the linea aspera were used as reference landmarks. Multiple iterations of the cutting guide were produced (Table 1), before settling on the third iteration, which was tested on a sawbone femur. The total length of the longitudinal cut of the planned osteotomy for the sawbone femur was 149.58 mm, while the actual osteotomy was 146.05 mm. The planned length of the transverse cut at the distal end of the sawbone femur osteotomy was 27.83 mm, while the actual was 22.23 mm wide. The percent error in length of the longitudinal and transverse cuts was found to be 2.36% and 20.12%, respectively. After the sawbone testing, the simplified and more robust fourth iteration was created with the goal of reducing the deviation from the planned procedure.

**Table 1:**
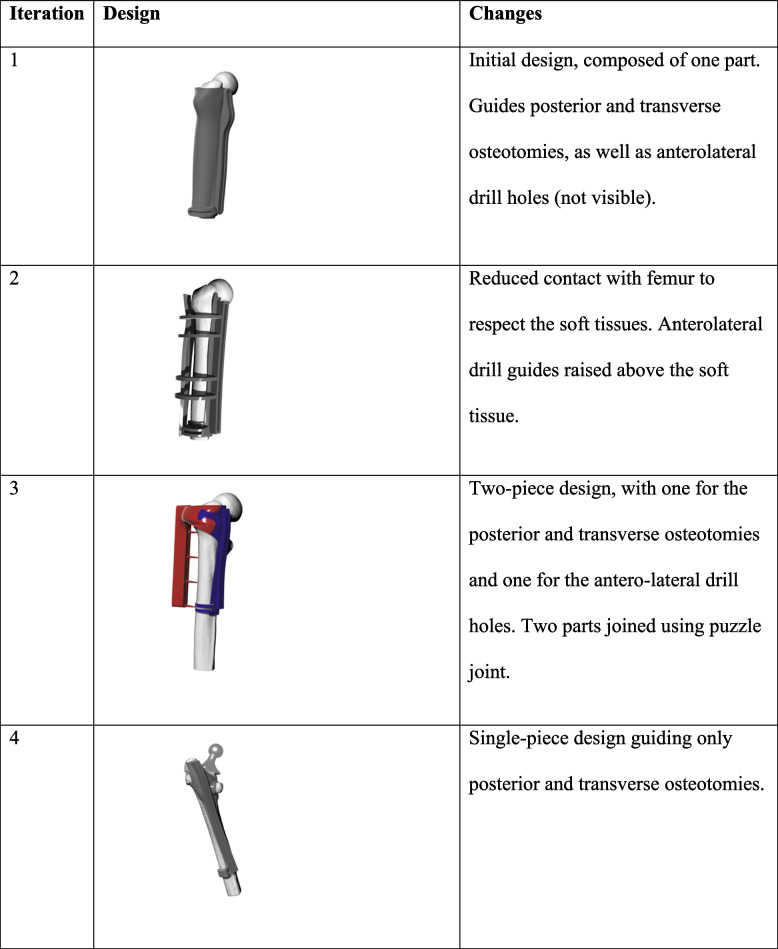
Design iteration

### Cadaver testing results

**Fig. 4:**
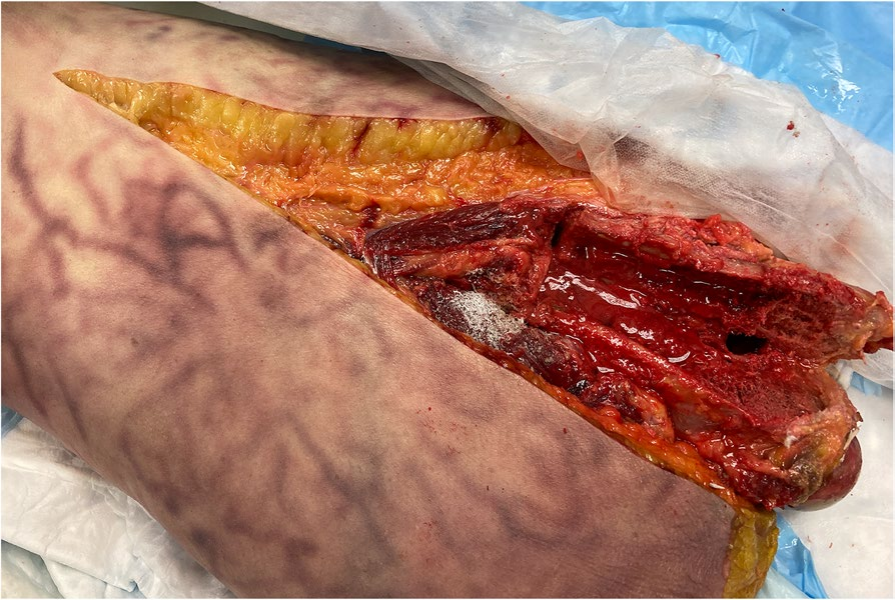
Final osteotomy of Cadaver 2 Left femur performed with the surgical guide

For all three cadaver femurs, the cutting guides enabled accurate positioning of the ETO, and allowed for visualization of the intramedullary canal and the implant. The cutting guides were able to accurately conform to the geometry of the femur and were fixed using K-wires (Fig. 3). Testing of the different levels of offset revealed that no additional offset was needed to accommodate for soft tissue in the fourth iteration for the guide to accurately conform to the femur, and it was noted that usage of the guide required no additional dissection or disruption of the soft tissue envelope, beyond that of a standard extended trochanteric osteotomy. Furthermore, the guide effectively aided the placement of the transverse and longitudinal osteotomies, exposing the entirety of the intramedullary canal and hip implant (Fig. 4) without causing any fractures of the osteotomy fragment and minimizing disturbance of soft tissue. Measurement of the osteotomies revealed that they deviated less than 6 mm from the planned osteotomy (Table 2).

**Table 2:** Error in actual osteotomy vs. planned osteotomy including Cadaver 1 (with Grey resin) and Cadaver 2 Right (with Biomed Amber resin) and Cadaver 2 Left (with Tough 2000 resin)

	**Longitudinal Osteotomy**			**Transverse Osteotomy**		
	**Planned Length (mm)**	**Actual Length** **(mm)**	**Percent Error** **(mm)**	**Planned Width** **(mm)**	**Actual Width** **(mm)**	**Percent Error** **(mm)**
*Cadaver 1*	144.25	143.0	0.86%	27.26	28.0	2.71%
*Cadaver 2 Left*	134.67	131.5	2.35%	12.46	13.3	6.86%
*Cadaver 2 Right*	141.22	136.6	3.27%	7.75	7.5	3.23%

The guide overall withstood use with the oscillating saw. There was no noticeable degradation of the interior slot of the guide, either from the cutting action of the blade or from the heat generated by the blade cutting through bone and cement mantle. All three resin used had a heat deflection temperature greater than 60 °C at 0.45 MPa [21–23]; no heat deflection was observed during use of the cutting guide. Some of the small features holding the two sides of the guide together sheared from the rapid vibrations of the saw, but did not appear to affect the function or accuracy of the cutting guide.

## Discussion

Our final iteration of the 3D-printed surgical guide functioned as designed, and was able to accurately conform to the femur. The guide enabled accurate osteotomies and respected the soft tissues, enabling visualization of the intramedullary canal and femoral implant. Testing of the final design on cadaveric femurs revealed that the vertical osteotomy had a maximum of 3.27% error in length, and the transverse osteotomy had a maximum of 6.86% error in width, compared to the planned osteotomy. This deviation is similar to other reported patient specific 3D surgical guides, such as 3D printed drill guides [15, 16] and cutting guides [14, 17]. These devices reported ~ 1-2 mm of deviation, comparable to the < 1 mm error in width experienced with our cutting guide. As the ETO is typically performed to 1 cm distal to the implant this degree of variation will adequately expose the entirety of the femoral implant. While maximum error in length reported with our cutting guide was greater (4.62 mm) than other 3D-printed PSI, our guide spanned a greater distance, and the overall percent error is therefore likely to be comparable.

Our method of iteration through different designs revealed several best practices for the development of future patient specific surgical guides. Most notably, our testing revealed the importance of simplicity in the design of the guide. More complex designs were more likely to be inaccurate and would frequently break during testing. The individual features of the guide had to be relatively small so that the overall guide would not be too bulky; however, this resulted in thin features too weak to withstand the load from the sagittal saw cutting through the femur. By simplifying the final design, we made the guide robust, compact, and effective. The cutting guide withstood use with an oscillating saw, with no observed degradation to the interior slot with which the blade interacted with. Some of the delicate features holding the two sides of the guide together sheared from the rapid oscillations of the saw, but did not appear to affect the guide function, and could likely be avoided by strengthening these features in future designs.

Through the design process, we found that it was important that the surgical guide did not encapsulate more than 180° of the femur. With Sawbones, a > 180^o^ encapsulation enabled the surgical guide to snap on firmly and stay fixed. However, on the cadavers, this feature made it difficult to accurately align the surgical guide with the femur and prevented easy attachment of the cutting guide. Rather, the K-wires were sufficient to maintain the positioning of the surgical guide, while the lesser trochanter and linea aspera were sufficient to initially position and align the guide on the femur.

The extended trochanteric osteotomy is an effective technique for rTHAs, enabling improved visualization and access to the femoral canal and femoral implant and aiding in the removal of well-fixed implants and cement. It is nonetheless a challenging procedure, requiring an experienced surgeon. A patient specific cutting guide may simplify the procedure, reduce operative time, prevent surgical errors, improve the likelihood of bone healing and trochanteric escape, and may provide a less experienced surgeon confidence to perform an ETO. This study describes a workflow for developing patient specific cutting guides to aid in ETOs resulting in precise osteotomies, within millimeter accuracy to pre-planned virtual procedures based on pre-operative CT imaging. The overall cost for these guides is low; the guides require less than 50 ml of resin (equivalent to around 15 USD). The engineering labor cost, including oversight of the 3D printer, is approximately 900 USD at our institution (6 h at USD150 per hr), With an automated image segmentation and CAD workflow, these labor costs could be reduced. The preoperative CT imaging is estimated to cost between 200 to 500 USD. We thus estimate the total cost, including imaging, to be under 1500 USD. Ultimately, we believe the value provided by the guide, with improved procedural accuracy to be worth the cost.

While this study has primarily discussed the customization of these guides to patient anatomy, it is worth noting the guides may be customized to individual surgeon preferences. For example, a surgeon may prefer to bevel the distal cut, or to use a pencil burr rather than the sagittal saw as described in this study. Simple changes to the geometry used to model the osteotomies can allow a surgeon to customize the procedure to their surgical preferences.

### Limitations

Cadaveric testing is inherently limited by the individual characteristics of each femur. Implants used for the primary arthroplasty vary in design and dimension, and have different modes of fixation (cement, amount of ingrowth, regions of ingrowth). These individual factors not only show the inherent need for personalized ETO guides, but also make generalization of the workflow discussed in this paper challenging. While the cutting guides were successful in guiding the osteotomy in the three cadaveric tests, these tests may not represent all cases. Only one of the three cadaveric femurs had an implanted stem, and thus this study may not represent the full diversity of stem geometry, as well as their potential to introduce metal artifacts into the CT imaging, both of which may affect the efficacy of the guide. Due to cost constraints and ethical considerations, first generations were developed with sawbone models, and then findings were extended to more developed and advanced models for cadaveric testing. Regardless, further trials are needed to evaluate the effectiveness of the surgical guide in a wider range of cases. However with personalized guides, the utilization of cadavers is a reasonable surrogate as there is nothing that the actual patient could present with that would be different than the cadaver would provide. Additionally, the ETO performed on the femur with the implanted stem had the least deviation from the planned ETO, suggesting that the presence of metal artifacts did not substantially affect the accuracy of the cutting guide. One additional limitation is that alternate technologies, such as intraoperative computer navigation, may be able to assist with ETOs but requires future investigation.

## Conclusion

The final 3D printed custom ETO cutting guide design was accurate in guiding the osteotomy within a few millimeters of error compared to the virtual planned osteotomy. In our iteration through multiple designs, we discovered the importance of simplicity for a robust and precise cutting guide. While further testing in clinical trials is needed, this study illustrates the potential of patient specific 3D-printed cutting guides to improve ETOs and their outcomes.

## Data Availability

The datasets used and/or analyzed during the current study are available from the corresponding author on reasonable request.

## Footnotes

1 ETO: Extended Trochanteric Osteotomy. rTHA: Revision Total Hip Arthroplasty. PSI: Patient Specific Instrumentation.

## References

[1] Peters PC, Head WC, Emerson RH (1993). An extended trochanteric osteotomy for revision total hip replacement. J Bone Joint Surg British.

[2] Younger TI (1995). Extended proximal femoral osteotomy: a new technique for femoral revision arthroplasty. J Arthroplasty.

[3] McGrory BJ, Bal BS, Harris WH (1996). Trochanteric osteotomy for total hip arthroplasty: six variations and indications for their use. JAAOS-J Am Acad Orthopaed Surg.

[4] Miner TM (2001). The extended trochanteric osteotomy in revision hip arthroplasty: a critical review of 166 cases at mean 3-year, 9-month follow-up. J Arthroplasty.

[5] Wronka KS (2020). Extended trochanteric osteotomy: improving the access and reducing the risk in revision THA. EFORT Open Rev.

[6] Huffman GR, Ries MD (2003). Combined vertical and horizontal cable fixation of an extended trochanteric osteotomy site. JBJS.

[7] Sambandam SN (2016). Extended trochanteric osteotomy: current concepts review. Eur J Orthop Surg Traumatol.

[8] Mardones R (2005). Extended femoral osteotomy for revision of hip arthroplasty: results and complications. J Arthroplasty.

[9] Bagaria V, Chaudhary K (2017). A paradigm shift in surgical planning and simulation using 3Dgraphy: experience of first 50 surgeries done using 3D-printed biomodels. Injury.

[10] Bernhard J-C (2016). Personalized 3D printed model of kidney and tumor anatomy: a useful tool for patient education. World J Urol.

[11] Di Laura A (2020). Can 3D surgical planning and patient specific instrumentation reduce hip implant inventory? A prospective study. 3D Print Med..

[12] Wong KC (2016). 3D-printed patient-specific applications in orthopedics. Orthop Res Rev.

[13] Baraza N (2020). 3D - printed patient specific instrumentation in corrective osteotomy of the femur and pelvis: a review of the literature. 3D Print Med.

[14] Nizam I, Batra AV (2018). Accuracy of bone resection in total knee arthroplasty using CT assisted-3D printed patient specific cutting guides. Sicot-J.

[15] Raaijmaakers M (2010). A custom-made guide-wire positioning device for hip surface replacement arthroplasty: description and first results. BMC Musculoskelet Disord.

[16] Roytman GR (2022). Accuracy of guide wire placement for femoral neck stabilization using 3D printed drill guides. 3D Print Med.

[17] Zakani S (2021). Computer-assisted subcapital correction osteotomy in slipped capital femoral epiphysis using individualized drill templates. 3D Print Med.

[18] Fan H (2015). Implantation of customized 3-D printed titanium prosthesis in limb salvage surgery: a case series and review of the literature. World J Surg Oncol.

[19] Wong KC (2015). One-step reconstruction with a 3D-printed, biomechanically evaluated custom implant after complex pelvic tumor resection. Comput Aided Surg.

[20] Li B (2018). Clinical value of 3D printing guide plate in core decompression plus porous bioceramics rod placement for the treatment of early osteonecrosis of the femoral head. J Orthop Surg Res.

[21] Formlabs, Standard Resins - Technical Data Sheet. 2023.

[22] Formlabs, BioMed Amber - Technical Data Sheet. 2023.

[23] Formlabs, Tough 2000 - Technical Data Sheet. 2020.

